# Low prevalence of p16-positive HPV-related head-neck cancers in Thailand: tertiary referral center experience

**DOI:** 10.1186/s12885-019-6266-0

**Published:** 2019-11-06

**Authors:** Titaporn Nopmaneepaisarn, Napadon Tangjaturonrasme, Worawat Rawangban, Chanida Vinayanuwattikun, Somboon Keelawat, Andrey Bychkov

**Affiliations:** 1 Department of Pathology, Faculty of Medicine, Chulalongkorn University, Bangkok, 10330 Thailand; 2 Department of Otolaryngology, Faculty of Medicine, Chulalongkorn University, Rama IV Rd., Pathumwan, Bangkok, 10330 Thailand; 3 Department of Internal Medicine, Faculty of Medicine, Chulalongkorn University, Bangkok, 10330 Thailand; 4 The King Chulalongkorn Memorial Hospital, Bangkok, 10330 Thailand; 5 Department of Pathology, Kameda Medical Center, Kamogawa, Chiba, 296-8602 Japan; 6 Department of Pathology, Nagasaki University Graduate School of Biomedical Sciences, Nagasaki, 852-8523 Japan

**Keywords:** Human papillomavirus (HPV), p16, Head and neck cancer, Squamous cell carcinoma, Oropharyngeal cancer, Laryngeal cancer, Oral cancer, Thailand, Epidemiology

## Abstract

**Background:**

There has been a sharp rise in the incidence of human papillomavirus (HPV) associated oropharyngeal squamous cell carcinoma (OPSCC) in many countries. Patients with HPV-positive OPSCC have a more favorable prognosis compared with HPV-negative OPSCC, leading to investigation and adoption of de-escalation treatment protocols. The baseline rate of HPV prevalence in certain populations is of epidemiologic significance. We aimed to evaluate the rate of high-risk HPV in a large cohort of Thai patients, including OPSCC, oral SCC (OSCC) and laryngeal SCC (LSCC).

**Methods:**

In total, 504 patients with HN cancer (110 OPSCC, 260 OSCC and 134 LSCC) who had been treated in Chulalongkorn University between 2010 and 2016 formed the sample set. All histological slides were reviewed to validate the diagnosis and render the histological type as keratinizing (K), non-keratinizing (NK) or non-keratinizing with maturation (NK-M). Immunohistochemistry with p16 was performed in all cases and scored semiquantatively. Positive and equivocal cases were tested by the high-risk HPV DNA in situ hybridization (ISH). Validation with quantitative polymerase-chain reaction (qPCR) was performed in p16-positive OPSCC.

**Results:**

The OPSCC were represented by NK (7.3%), NK-M (16.4%) and K (76.4%) types, with an HPV incidence of 100, 22.2 and 4.7%, respectively. The average HPV prevalence in OPSCC was 14.5%. The concordance with p16/ISH was 51.6%, while concordance of the NK morphology with positive HPV ISH was 100%. ISH-qPCR concordance in p16-positive OPSCC was 72.7%. Patients with HPV-positive OPSCC had significantly more tumors with a NK histologic type, tonsillar location, earlier clinical stage, less association with smoking, and, finally, better outcome and longer survival time. In non-OPSCC, p16-positive HPV-associated cancers were found in only 1.5% of OSCC (4/260) and LSCC (2/134).

**Conclusion:**

A low rate of HPV-related OPSCC was found in Thai patients. The NK morphology was an excellent predictor of high-risk HPV infection in OPSCC. For OPSCC patients, HPV-positive ones had a significantly longer survival time than HPV-negative ones. There was a lack of p16-positive HPV-related OSCC and LSCC. Morphology and p16 status had a poor predictive value for detecting HPV in OSCC and LSCC.

## Background

Head and neck (HN) squamous cell carcinoma (SCC; HNSCC) is one of the most common cancers worldwide [1]. Oral cavity, larynx and oropharynx are the major anatomic locations of SCC of the upper aerodigestive tract [2]. The well-known risk factors include tobacco smoking, alcohol consumption [3] and oral betel quid use [4]. Transcriptionally-active high-risk human papillomavirus (HPV) was found to be associated with a subset of HN cancers [1]. Numerous studies on HPV-related HNSCC reported that the prevalence of HPV-related HNSCC is quite high, especially for the oropharyngeal region [5, 6].

The HPV-positive oropharyngeal SCC (OPSCC) has unique clinical characteristics and tumor morphology [1, 7]. Compared to HPV-negative cancers, the HPV-positive OPSCCs tend to have a better survival outcome [8, 9]. These tumors typically arise in younger patients, especially in males with sexual-risk behaviors and no significant history of tobacco smoking, alcohol consumption or oral betel quid use [7]. Regarding the tumor morphology, HPV-associated HN cancers display non-keratinizing features, which is currently accepted as the so-called “prototypical HPV-related morphology” [7, 10].

For OPSCCs, there are recommendations that detection of transcriptionally-active HPV should be performed as a routine pathology practice [7, 11]. There are several HPV detection methods, including p16 immunohistochemistry, DNA in situ hybridization (ISH), RNA ISH, polymerase chain reaction (PCR) for HPV DNA and PCR for E6/E7 mRNA. Each technique has their benefits and disadvantages [11]. The most common multimodality method considers performing p16 immunostaining as a screening step followed by a virus-specific test, which could be either HPV type-specific DNA PCR or ISH [11, 12]. Interestingly, recent studies found that p16 expression by OPSCC combined with prototypical HPV-related morphology was sufficient to diagnose it as HPV-positive [7, 13].

A contemporary meta-analysis revealed a very high prevalence of HPV association with OPSCC in North America (69.7%) and Europe (73.1%), while non-OPSCC had much lower rates of HPV (21.8%) [14]. The most recent publications estimated that HPV-associated OPSCC is approaching 80% in the United States and still rising [1]. The prevalence of HPV in HN cancers from Southeast Asia in general, and Thailand in particular, is not well studied. Available publications suggest a much lower prevalence level compared to the Western cohorts; about 17% in OPSCC [15, 16], 23–48% in laryngeal cancer [6] and 3–30% in oral cavity cancer [15–18]. However, most of these studies could not be considered reliable and representative due to their limited sample size and wide array of tissue samples (e.g., saliva and mucosal swabs) and techniques used.

We believe that a large-scale study performed in the settings of a tertiary referral center for HN cancer will provide robust numbers on the HPV prevalence in the local cohort of HNSCC. These data are of epidemiological significance and may have potential impact for preventive and therapeutic strategies at a national level. For example, prophylactic vaccination could be of public health benefit in the prevention of HPV-associated cancer in countries with a high regional incidence, which is currently widely accepted for cervical cancer [19, 20]. In this study, we aimed to evaluate the prevalence of high-risk HPV in Thai HNSCCs, including true HPV-associated (OPSCC) and potentially HPV-related (laryngeal and oral) cancers, based on a two-step approach combining p16 immunostaining and HPV DNA ISH.

## Methods

### Study cohorts

A retrospective search (January 2010 to December 2016) of all the cases of OPSCC, oral SCC (OSCC) and laryngeal SCC (LSCC) was performed in the electronic database of the Department of Pathology and HN Cancer Tumor Board database, King Chulalongkorn Memorial Hospital, Bangkok. The inclusion criteria were pathologically confirmed SCC and availability of the paraffin-embedded tissue blocks. Non-invasive tumors (carcinoma in situ), cancers other than pure SCC (e.g., verrucous, adenosquamous and undifferentiated carcinoma, etc.), and undoubtedly metastatic SCCs from other regions were all excluded. Searchable clinical parameters were recorded, including the age, gender, sampling procedure, TNM category, clinical stage, treatment modality, smoking and alcohol consumption status, follow up duration, oncological outcome and survival status. In addition, OPSCC patients were classified into low-, intermediate- and high-risk groups based on the stratification algorithm by Ang et al. [8]

This study was approved by the Institutional Review Board of the Faculty of Medicine, Chulalongkorn University (Certificate of Approval CoA 531/2016). The need for consent was waived by the Institutional Review Board.

### Histopathological evaluation

**Fig. 1:**
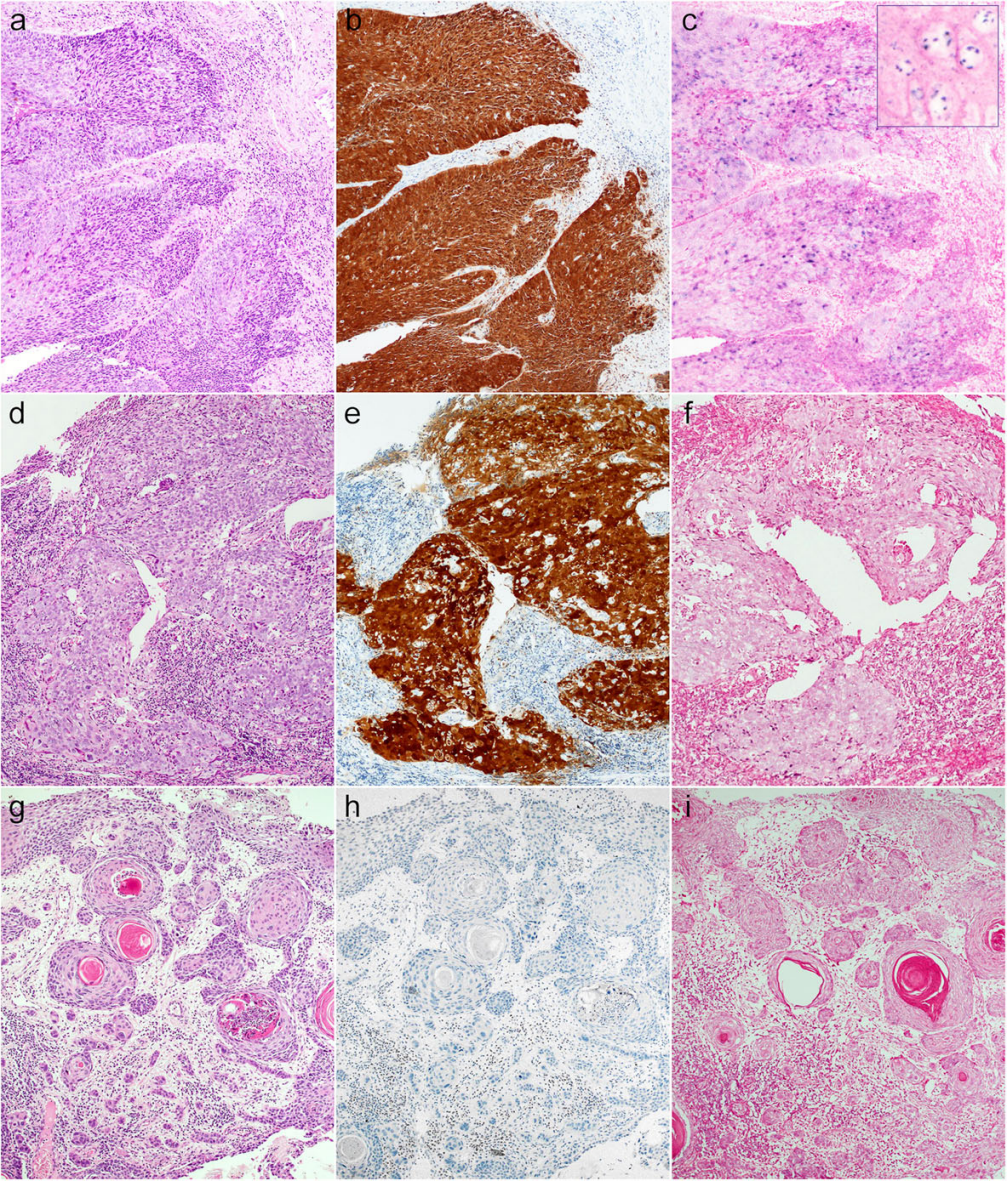
Representative cases of NK, NK-M and K histological subtypes of OPSCC K cancer with sheets of compact cells (**a**), which are p16-positive (**b**) and HPV DNA ISH-positive (**c**). Inset shows intranuclear localization of HPV consistent with so-called “integrative” pattern of viral infection. NK-M case (**d**) is predominantly composed of compact cells mixed by areas with larger cells with more abundant eosinophilic cytoplasm (lower left); NK-M OPSCC are often p16-positive (**e**), but HPV ISH-negative (**f**). K carcinoma with typical keratin pearls (**g**), no p16 immunoreactivity (**h**), and no HPV ISH signal. Hematoxylin & eosin (**a**, **d**, **g**), p16 immunohistochemistry (**b**, **e**, **h**), high-risk HPV DNA ISH (**c**, **f**, **i**); semi-serial sections, × 100.

All histological slides were reviewed to validate the original diagnosis and render the morphologic type of OPSCC, as keratinizing (K), non-keratinizing (NK) or non-keratinizing with maturation (NK-M), or grade of OSCC/LSCC, as well-, moderately- and poorly-differentiated. The NK morphology was defined as sheets, nests or trabeculae of tumor cells with oval-to-spindle-shaped, hyperchromatic cells, high nuclear-cytoplasmic ratio, indistinct cell borders and inconspicuous nucleoli, added by frequent comedo-type necrosis and a high mitotic index (Fig. 1a) [21]. The K type was assigned to tumors without areas of NK morphology, with totally maturing squamous differentiation or squamous maturation present throughout the tumor [21]. Squamous maturation was characterized by polygonal-shaped cells with abundant eosinophilic cytoplasm, distinct cell borders, intercellular bridges and keratin pearls (Fig. 1f). The NK-M type consisted of features of both types, where a definite NK morphology had to be noted along with squamous maturation in more than 10% of the total tumor surface area (Fig. 1d) [21]. Squamous maturation in less than 10% of the total tumor surface area was allowed for inclusion to the NK type [21]. Tumor grade was defined as per the 2017 WHO Classification of HN Tumors [22].

### Immunohistochemistry and in situ hybridization

Whole mount sections of all the enrolled specimens (*n* = 504) were stained for p16 immunohistochemistry as a surrogate marker of HPV infection using a monoclonal antibody to p16 (CINtec® Histology, Ventana, Tucson, AZ) on a VENTANA BenchMark ULTRA instrument (Ventana, Tucson, AZ). A recently described “multiple sections per slide” technique was applied [23]. Immunostaining of p16 was scored as positive (Fig. 1b, e), equivocal or negative (Fig. 1h), if nuclear and cytoplasmic staining was observed in > 70%, 30–70% or < 30% of the cancer cells, respectively. Initially, only p16-positive and equivocal cases were tested by DNA ISH HPV III Family 16 Probe (Ventana Medical Systems, Tucson, AZ) specific for high-risk types HPV, including types 16, 18, 31, 33, 35, 39, 45, 51, 52, 56, 58, and 66. Second run of HPV ISH was performed on all p16-negative OPSCC and 125 random p16-negative OSCC samples. The HPV ISH was considered positive when episomal and/or integrative patterns were noted within the cancer cells [24]. The episomal pattern was defined as large homogeneous round navy-blue probes occupying the nucleus. The integrative pattern was represented by smaller discrete navy-blue probes imprinting the cancer nuclei (Fig. 1c, inset).

### HPV DNA genotype detection

According to the standard protocol, 10 sections of 10 μm thick paraffin-embedded tissue were microdissected from selected tissue blocks after matching with histological slides. DNA was extracted using QIAamp FFPE tissue extraction kit (Qiagen, Hilden, Germany) following the manufacturer’s protocol and stored at − 20 °C until used. Only specimens with adequate amount of DNA were further analyzed using quantitative real-time PCR-based detection testing (qPCR) on Qubit 2.0 fluorometer (Thermo Fisher Scientific, CA, USA) and Qubit® dsDNA HS Assay Kit (Life Technologies, Thermo Fisher Scientific, CA, USA). Hundred nanograms of extracted DNA was used as an input for HPV genotype testing using AmoyDx® Human Papillomavirus Genotyping Detection Kit (Amoy Diagnostics, China) following the manufacturer’s protocol. This kit covered 19 high-risk HPV types (HPV16, 18, 26, 31, 33, 35, 39, 45, 51, 52, 53, 56, 58, 59, 66, 68, 70, 73, and 82) and 2 low-risk HPV types (HPV6 and 11). The kit was composed of 22 HPV DNA positive reference controls, and 5 negative reference controls which allowed 100% accuracy for concordance detection rate with minimal detection of 100 copies HPV DNA per reaction. Multiple blanks were incorporated in PCR reactions to exclude cross-contamination. All qPCRs were conducted in duplicate fashion using ABI 7500 sequence detection analyzer (Thermo Fisher Scientific, CA, USA).

### Statistical analysis

Statistical analysis was performed using the t-test, U-test and Chi-square. Survival analysis was conducted for the outcome of overall survival, with time to the outcome calculated from the date of diagnosis. Patients without events were censored at the date of last known follow-up. Unadjusted survival curves were obtained using Kaplan-Meier estimates and compared with the log rank test. Two-sided *P* value less than 0.05 was considered statistically significant. Graphical representation, statistical and survival analysis were performed in GraphPad Prism 6 software (GraphPad, La Jolla, USA).

## Results

### Oropharyngeal cancer cohort

A total of 110 cases of OPSCC were enrolled (Table 1). Most of the patients were male (*n* = 95) with a 6.3:1 male-to-female ratio. The mean age was 59 years (range, 28–89 years). Tissue specimens were mainly from tumor biopsies (*n* = 94) with a small number of surgical samples (*n* = 16). The 110 cases of OPSCC were histologically represented by NK (eight cases, 7.3%), NK-M (18 cases, 16.4%) and K (84 cases, 76.4%) subtypes. Immunohistochemistry for p16 showed positive results in seven NK, 10 NK-M and 14 K cases (Table 1, Additional file 1). Then, HPV DNA ISH was performed on all OPSCC cases, and showed a positive signal in eight (100%), four (22%) and four (4.7%) cases from the NK, NK-M and K subtypes, respectively. Overall HPV prevalence evaluated by ISH in the OPSCC cohort was low at 14.5%. The p16 and HPV DNA ISH results are presented in Table 2 and illustrated in Fig. 1. Concordance between p16 immunohistochemistry and HPV DNA ISH in the OPSCC samples was 51.6%. Of note, all of the OPSCC cases of the NK type were HPV-positive (100%).

**Table 1:** Clinicopathological characteristics of patients from the oropharyngeal cancer cohort (*n* = 110)

	n	%
Male: Female	95:15 (6.3:1)	
Age, mean (range)	59 (28–89)	
Tumor sampling		
Surgery	16	14.5%
Biopsy	94	85.5%
Site		
Base of tongue	43	39.1%
Tonsil	36	32.7%
Pharyngeal wall	14	12.7%
Soft palate	17	15.5%
Squamous cell carcinoma type:		
Non-keratinizing	8	7.3%
Non-keratinizing with maturation	18	16.4%
Keratinizing	84	76.4%
p16 status		
p16-positive	31	28.2%
p16-negative	79	71.8%
HPV status		
HPV-positive	16	14.5%
HPV-negative	94	85.5%
Risk-of-death categories		
Low risk	18	18.4%
Intermediate risk	21	21.4%
High risk	59	60.2%
Treatment		
RT + systemic	63	57.3%
RT	16	14.5%
Surgical + chemoradiotherapy	9	8.2%
Palliative RT/CCRT	4	3.6%
No data	18	16.4%
Follow-up status		
Alive	42	38.2%
Death	68	61.8%
Survival (months)		
Mean	23.1	
Median	17	
Median follow-up (months)		
Alive group	30.5 (14–90)	
Died group	10 (1–53)	

*HPV* human papilloma virus, *RT* radiotherapy, *CCRT* concurrent chemoradiotherapy

**Table 2:** Concordance between histological type of OPSCC and ancillary testing for high-risk HPV

Tumor type	n	p16+	ISH+	qPCR+^a^
Keratinizing	84	14	4	5/9
Non-keratinizing with maturation	18	10	4	5/8
Non-keratinizing	8	7	8	5/5
Total	110	31	16	15/22

^a^ only p16-positive cases with adequate DNA yield were tested by qPCR

*ISH* DNA in situ hybridization, *qPCR* quantitative polymerase chain reaction

### Validation of ISH by qPCR in OPSCC

To validate results of ISH, we performed HPV DNA genotype detection with qPCR in all p16-positive specimens (*n* = 31). DNA amplification was successful in 22 samples. Sixteen cases showed agreement between ISH and PCR results (Table 2). Among 6 discordant samples, five ISH-negative cases turned to be HPV-positive by PCR while one case strongly positive by ISH produced negative PCR result. Thus, qPCR was more sensitive in detection of high-risk HPV in our OPSCC cohort. The concordance between ISH and PCR was 72.7%. Considering such relatively high ISH-qPCR concordance, and for consistency (we were able to perform ISH but not qPCR in all specimens), ISH result was chosen as a ground truth for HPV status of the tumor.

### Outcome in OPSCC patients

Due to the advanced stage of the disease, 63 cases with OPSCC received radical radiation combined with systemic therapy (chemotherapy or targeted therapy) and 16 cases received radical radiation alone. Only nine cases were suitable for upfront surgery with post-operative radiation. In the OPSCC cohort, 42 patients (38.2%) were alive with a median 30.5 months of follow up period and 68 patients (61.8%) died with a median 10 months follow up (Table 1).

**Fig. 2:**
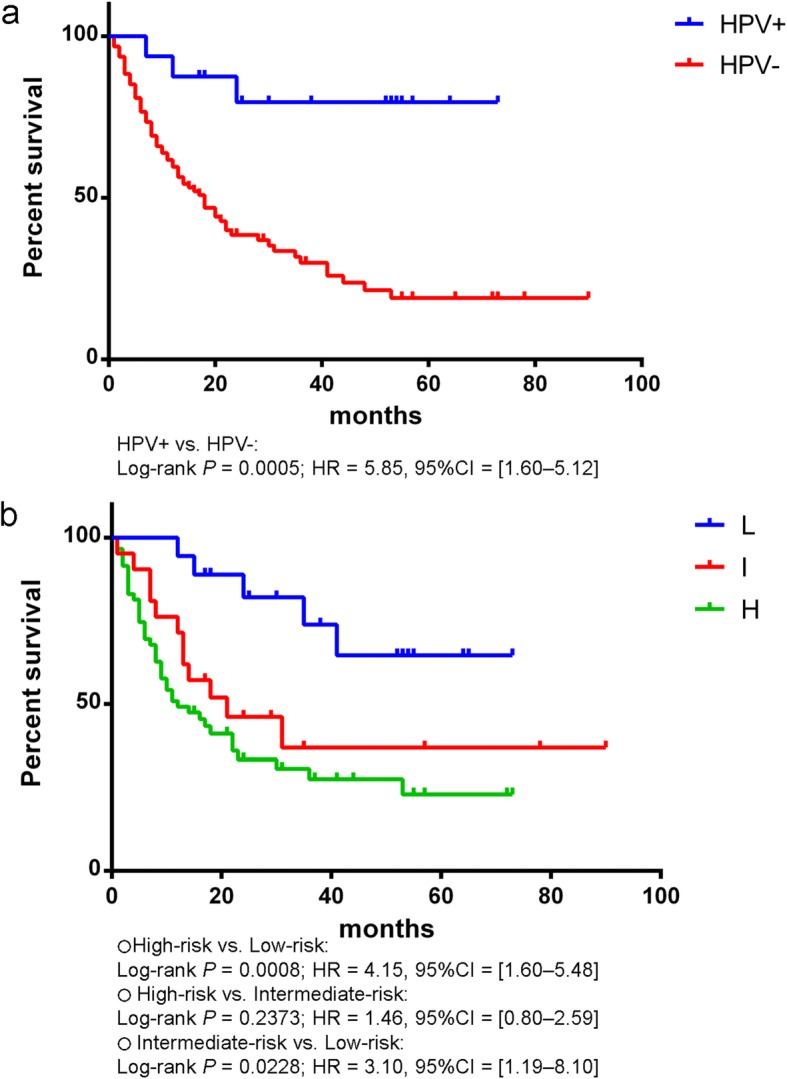
Survival of patients with OPSCC. HPV-positive patients had significantly better survival compared to HPV-negative ones (**a**). OPSCC patients stratified by risk-of-death into low-, intermediate- and high-risk groups (**b**)

Subgroup analysis between ISH-positive and ISH-negative OPSCC found a significantly better outcome in patients with HPV-associated cancer, including a lower mortality and longer survival (Fig. 2a). From the survival analysis, a significant difference between ISH-positive and ISH-negative OPSCC cohorts (*p* = 0.0005, Log rank test) was evident with a hazard ratio of 5.85 (95% confidence interval = 1.60 to 5.12). In addition, the cohorts showed a difference in their NK morphology, tonsil location, the early stage of disease and lack of smoking history (Table 3). Concurrent chemoradiation was the main treatment modality in both groups (68.7 and 57.1%, HPV-positive vs. HPV-negative) followed by radical radiation alone (12.5 and 22.6%) and surgical treatment (12.5 and 8.3%). The risk-of-death stratification based on the combination of HPV status, smoking history, tumor and nodal stage [8] classified all OPSCC patients into low-, intermediate- and high-risk groups (Fig. 2b). There were 18, 21 and 59 patients in each group, respectively. We found a statistically significant difference in survival between low-risk and intermediate-risk groups (*p* = 0.03), and between low-risk and high-risk groups (*p* = 0.01), but not between intermediate-risk and high-risk groups (*p* = 0.24).

**Table 3:** HPV-positive vs. HPV-negative OPSCC

	HPV+ (*n* = 16)		HPV- (*n* = 94)		*p*-value
	n	%	n	%	
Mean age (years)	59.1		59.0		0.98
M:F ratio	4.3:1		6.8:1		0.52
NK morphology	8	44%	0	0%	< 0.001^a^
K morphology	5	28%	79	86%	
p16-positive	15	93.75%	16	17%	< 0.001^a^
Tonsil	13	81.25%	24	25.53%	< 0.001^a^
Alive	13	81.25%	29	30.85%	< 0.001^a^
Median survival (months)	34		15		0.03^a^
T1–2 stage	12	75%	34	36.17%	0.006^a^
N1–3 stage	13	81.25%	66	70.21%	0.55
M1 stage	0	0%	6	7%	0.26
Smoking	8/16	50%	68/85	80%	0.01^a^
Alcohol	8/12	67%	54/74	74%	0.73

^a^ denotes statistically significant difference

*HPV human papilloma virus*, *NK* non-keratinizing, *K* keratinizing

### Oral cancer and laryngeal cancer cohorts

Baseline characteristics of the OSCC and LSCC cohorts are described in Table 4 and Additional file 1. There were only nine OSCC (3%) and six LSCC (4%) samples that were positive for p16. Two-step testing with p16 immunohistochemistry followed by HPV DNA ISH for the positive and equivocal cases found p16-positive HPV-associated cancer in a very low number of OSCC (4/260, 1.5%) and LSCC (2/134, 1.5%) cases. Concordance with p16-ISH was less than 50% for both locations. Furthermore, the NK morphology was rare in LSCC (6.7%) and exceedingly rare in OSCC (1.5%) and, in contrast to OPSCC, was not associated with p16-positivity and HPV ISH detection. With such a very low incidence of p16-positive HPV-associated cases, we did not perform further subgroup analysis in the OSCC and LSCC cohorts. To validate an exceedingly low rate of HPV in OSCC, we performed additional HPV DNA ISH testing in 125 cases of p16-negative OSCC with available follow up. None of them turned out to be HPV DNA-positive.

**Table 4:** Baseline characteristics of patients with oral and laryngeal SCC

	Oral SCC (*n* = 260)	Laryngeal SCC (*n* = 134)
Male : Female	151:109	127:07
Age (years)		
Mean	61.3	63.4
Range	29-95	36-89
Surgery : Biopsy	133:127	49:85
Site		
	lip, *n* =18	supraglottic, *n* = 34
	oral tongue, *n* =159	glottic, *n* = 6
	floor mouth, *n* = 27	subglottic, *n* = 3
	buccal, *n* = 21	transglottic, *n* = 71
	alveolar ridge, *n* =18	not specified, *n* = 20
	hard palate, *n* = 8	
	retromolar trigone, *n* = 9	
Grade		
Well-differentiated	149	57
Moderately-differentiated	105	68
Poorly-differentiated	6	9
p16-positive	9	6
HPV ISH-positive	10*	2**
p16+/HPV ISH+	4 (1.5%)	2 (1.5%)

* out of 156 tested with ISH

** out of 7 tested with ISH

*SCC* squamous cell carcinoma, *HPV* human papilloma virus, *ISH* DNA in situ hybridization

## Discussion

In this large-scale study, we found that 14.5% of OPSCC were associated with a high-risk HPV. This low baseline rate of HPV-related OPSCC detected by the two-step approach of p16 immunohistochemistry followed by HPV DNA ISH was established in the Thai population for the first time. We also validated ISH results by qPCR in a subset of p16-positive OPSCC and found the concordance between two tests as high as 72.7%. The NK morphology of OPSCC was a strong predictor of high-risk HPV infection.

Our findings are consistent with the Asian data indicating that the prevalence of HPV-associated OPSCC is much lower than that in the Western countries. Thai patients with OPSCC had a lower HPV infection rate compared to that reported in South Asia (25.8%) and Eastern Asia (38.7%), and a considerably lower rate than in well-developed Asian countries, such as Japan (40.8%) and Korea (69.2%) [6]. At the same time, Western authors have reported an emerging HPV-induced epidemic of OPSCC in Europe and North America, which is characterized by a sharp increase in incidence over the last few decades (almost 10-fold), currently exceeding 80% prevalence of HPV in OPSCC [1]. Differences in the incidence of HPV-related OPSCC between Asian and Western countries might be a result of exposure to different risk factors. In general, HPV-positive cancer is associated with high-risk sexual behavior, whereas tobacco smoking, alcohol consumption, or oral betel quid use are risk factors of HPV-unrelated SCC [7]. Some local habits in Southeast Asian populations, including Thais, such as betel chewing or traditional cigar smoking, may have an etiologic role in certain cases of HPV-negative OPSCC [16].

There are no available reports on HPV-associated OPSCC in Thai patients published in international journals. Within the local literature only three studies on this topic were available [15, 25, 26]. All of them enrolled less than 35 samples and used a different methodology. Boonmark et al. showed an approximately similar low prevalence of HPV (17%, 5/30) based on detection of HPV DNA by PCR [15]. Two earlier studies found a 47–49% HPV rate [25, 26], but these results should be treated with caution. For instance, Petcharapirach et al. claimed HPV prevalence based only on p16 immunostaining without further confirmation by PCR or ISH [25], which would likely overestimate the HPV rate. For instance, there were 28.2% p16-positive OPSCC in our cohort, but only 16.4% turned to be true HPV-positive. Another study enrolled only patients of less than 45 years old, which could not be considered as a representative cohort [26], because HPV has a strong predilection to young patients. We believe that our study employed the most reliable approach to detect transcriptionally-active high-risk HPV in OPSCC and, therefore, presented an actual baseline rate of HPV-related OPSCC in Thailand.

Clinical correlation in the OPSCC cohort found that patients with HPV-related OPSCC shared similar (less aggressive cancer) clinical behavior with those reported from the regions with a high incidence of HPV-related OPSCC [8, 9]. Such characteristics included tonsillar area preference, frequent presentation with early stage disease, lower tobacco use, a predominant NK histology and a significantly better survival. Due to its nature of being a tertiary HN cancer center, the distribution of disease staging in our samples was mostly of the advanced stage in all the subgroups. As a result, up to two-thirds of the patients died of disease within 1–2 years after cancer diagnosis. In addition to the tertiary center bias, this could be explained by the low awareness of the local population about the early symptoms of HN cancers. Recently, we reported that in addition to the growing incidence, survival rates of HN cancers in Thailand are not improving over the years [16].

Proper identification of HPV-associated OPSCC is clinically relevant. Patients with HPV-positive OPSCC have a more favorable prognosis compared with the HPV-negative subtype, which may require less aggressive treatment. The most common approach for HPV testing in current practice is utilizing p16 immunohistochemistry, followed by either qPCR or ISH to detect HPV DNA or RNA [7, 11, 27–29]. Interestingly, recent studies found that a combination of p16 immunoexpression with the NK morphology is sufficient to diagnose OPSCC as HPV-positive [7, 13]. Our findings confirmed that the vast majority of NK OPSCC were p16-positive and HPV-positive (by both DNA ISH and qPCR). Nevertheless, there is a general agreement [11] that OPSCC with K and especially a NK-M and NK morphology require a further two-step algorithm for identification of their HPV-associated status. Immunostaining with p16 can serve as a screening test, which prompts PCR or ISH testing only for p16-positive samples of OPSCC.

Among 6 samples discordant by molecular testing, five ISH-negative cases turned to be HPV-positive by qPCR. This is not surprising because qPCR is more sensitive than DNA ISH for detection of high-risk HPV and still considered as a gold standard test [11]. We also compared a coverage of HPV types by both techniques and found that PCR kit was designed to detect 21 HPV genotypes vs. 12 genotypes by ISH. There was one case with strong positive result by ISH but remained negative after repeat qPCR testing. Clinical correlations (old age patient with heavy smoking and drinking habit) were rather pointing out to the phenotype of HPV-associated OPSCC. Finally, the concordance between ISH and PCR in our OPSCC series was 72.7%. Since we were not able to perform qPCR on all specimens due to the resource limitations and considering relatively high ISH-qPCR concordance, ISH result was chosen as a ground truth for HPV status of the tumor.

At variance with OPSCC, tumor histology and p16 had limited predictive value for high-risk HPV in OSCC and LSCC, and so we do not advocate the routine use of p16 immunohistochemistry as a surrogate marker for HPV infection in these cohorts. Consistent with Western reports, our study found a low concordance between p16 immunoexpression and the presence of HPV in non-OPSCC [30, 31]. The prevalence of p16-positive HPV-associated cancer in OSCC and LSCC from our cohort was exceedingly low at 1.5%. Of note, we are discussing here only a low prevalence of HPV associated with augmented p16 signaling in OSCC and LSCC, which was shown to be an oncogenic pathway in cervical cancer and OPSCC. The prevalence of HPV could be higher if we considered p16-negative high-risk HPV-positive cases and low-risk HPV-positive cases. However, the oncogenic role of HPV that is not associated with p16 overexpression is controversial. These translationally-inactive viruses are likely “passengers” that are not able to initiate cancer growth and, therefore, have no clinical significance on their own [30, 31].

This study had certain limitations that require further elaboration. Although HPV RNA ISH is currently considered as the gold standard for detection of transcriptionally-active HPV [5, 11, 13, 31], the major drawback of this technique is its high cost incompatible with routine practical workflow. Instead, we employed a well-validated two-step algorithm, which has proved efficient in numerous preceding studies and real-life scenarios [7, 11–13]. Furthermore, using qPCR validation, we found agreement between HPV ISH and qPCR in about three-fourths of p16-positive OPSCC cases. Hence, we verified a diagnostic algorithm for the detection of HPV-related OPSCC (i. e., p16 immunostaining followed by ISH) tailored to our settings. We did not perform HPV DNA ISH on all the samples from OSCC and LSCC cohorts, because the major focus was put on detecting cancer-initiating transcriptionally-active (i.e., p16-positive) HPV. The same reason could be applicable to ignoring the detection of low-risk HPV types, which require additional reagents.

## Conclusions

This is the first large scale study to date reporting a low rate of HPV-related OPSCC in Thailand (14.5%), which is consistent with Asian data. This reference rate of HPV prevalence is of epidemiological significance, and may have potential impact for national preventive and therapeutic programs. High-risk HPV infection was associated with the NK morphology of OPSCC. There was a lack of p16-positive HPV-related OSCC and LSCC, while immunoexpression of p16 had a poor predictive value for detecting HPV in OSCC and LSCC. Similar to in Western countries, Thai patients with HPV-positive OPSCC presented with an earlier stage, lack of smoking history and had a significantly longer survival time compared to those with HPV-negative OPSCC.

## Supplementary information

**Additional file 1.** Microsoft Office Excel with all the raw data relevant to the present study, including anonymized baseline, histological, and clinical variables.

## Data Availability

All data generated or analyzed during this study were anonymized and included in this published article and its supplementary information files (Additional file 1).

## Abbreviations

HN: Head and neck
HPV: Human papillomavirus
ISH: In situ hybridization
K: Keratinizing
LSCC: Laryngeal squamous cell carcinoma
NK: Non-keratinizing
NK-M: Non-keratinizing with maturation
OPSCC: Oropharyngeal squamous cell carcinoma
OSCC: Oral squamous cell carcinoma
PCR: Polymerase chain reaction
